# *Bacillus cereus* strains from donor human milk and hospital environment: uncovering a putative common origin using comparative analysis of toxin and infra-red spectroscopy profiles

**DOI:** 10.3934/microbiol.2023022

**Published:** 2023-05-04

**Authors:** Gaëtan Outurquin, Odile Obin, Anaïs Petit, Roxane Weiss, André Léké, Crespin Adjidé, Catherine Mullié

**Affiliations:** 1 Laboratoire Hygiène Risque Biologique & Environnement, Centre Hospitalier Universitaire Amiens-Picardie, Amiens, France; 2 Lactarium–Biberonnerie, Unité des soins intensifs de néonatologie et de médecine néonatale, Centre Hospitalier Universitaire Amiens-Picardie, Amiens, France; 3 Laboratoire AGIR UR UPJV 4294, UFR de Pharmacie, Université de Picardie Jules Verne, Amiens, France

**Keywords:** *Bacillus cereus*, cereulide toxin, pore-forming toxins, cytotoxin K, enterotoxins, Fourier-transform infra-red spectroscopy, human milk, human milk bank

## Abstract

*Bacillus cereus* is reported as a common cause of toxin-induced food poisoning and of contamination in pasteurized human milk donations. As various toxins can be produced by *B. cereus*, the aim of this work was first to investigate the toxigenic potential and profiles of 63 *B. cereus* isolates from Amiens Picardie human milk bank. A comparison to the toxigenic profiles of 27 environmental *B. cereus* isolates harvested in the hospital in which this human milk bank is situated was performed. Toxin gene prevalences were the highest for *nhe (ABC)* and *entFM* followed by *cytK* and *hbl(ACD)*. A 27% prevalence was found for *ces* human milk isolates, which is higher than previous works reporting on pasteurized milk and dairy products. No significant differences could be found between human milk and environmental isolates regarding toxin gene prevalences and/or toxin gene profiles. The second aim was to establish whether a *B. cereus* cross-contamination between human milk and the environment could occur. This was achieved with the help of Fourrier-transform infra-red spectroscopy which enabled the discrimination of 2 main clusters of 11 and 8 isolates, each containing human milk and Amiens Picardie human milk bank environmental isolates. For these two clusters, the time sequence showed that human milk isolates were the first to occur and might have contaminated the milk bank environment as well as other human milk donations. Routinely used on *B. cereus* isolates, Fourrier-transform infra-red spectroscopy could help in rapidly detecting such clusters and in limiting the spread of a *B. cereus* strain that might generate rejection of pasteurized donation by the human milk bank.

## 1. Introduction

Human milk stored in human milk banks (HMBs) is the gold standard for feeding preterm neonates, especially those born before 32 weeks of age. To reduce the infectious risk linked to a possible contamination by harmful bacteria through human milk of these preterm neonates, pasteurization (mainly standard Holder pasteurization at 62.5 °C for 30 min) is carried out in HMBs to kill bacteria present in raw human milk. However, *B*. *cereus* endospores may not completely be eliminated by pasteurization and can therefore give rise to new vegetative forms of *B. cereus* in the milk post-pasteurization [1]. Additionally, several reports show that pasteurized human milk can be contaminated with *Bacillus cereus* [2]–[4]. This is a cause for concern as preterm neonates have previously been reported to be susceptible to severe cases of *B. cereus* infections [5],[6] and human breast milk was pointed out as a possible source of contamination [5],[7]. The most usual symptoms of *B. cereus* infections are diarrheal and emetic [8]. Diarrheal symptoms are caused by a variety of toxins such as pore-forming toxins hemolysin BL (Hbl) and non-hemolytic enterotoxin (Nhe) which both comprise three subunits, as well as cytotoxin K (cytK) [8],[9]. Additionally, enterotoxins entFM and bceT, encoded respectively by *entFM* and *bceT*, have also been proposed to play a part in diarrheic symptoms witnessed in *B. cereus* food poisoning [10],[11]. However, the enterotoxic properties of bceT were later questioned [12]. Meanwhile, the emetic form of *B. cereus* infection is driven by the action of cereulide toxin, a cyclic dodecadepsipeptide resistant to heat, proteolysis and acidic pH [9]. The latter toxin is encoded by genes found on a megaplasmid and its prevalence in *B. cereus* strains is relatively low, as compared to other toxins [9],[13]. A classification of *B. cereus* strains in seven different toxin profiles: A (*nhe*+, *hbl*+, *cytK*+), B (*nhe*+, *cytK*+, *ces*+), C (*nhe*+, *hbl*+), D (*nhe*+, *cytK*+), E (*nhe*+, *ces*+), F (*nhe*+) and G (*cytK*+) has been proposed [14].

In Amiens-Picardie Human Milk Bank (APHMB), contamination rates with *B. cereus* of pasteurized milk have been shown to be the leading cause of milk rejection post-microbiological quality control [3]. Therefore, we endeavored to assess whether this contamination could (i) be due to *B. cereus* strains presenting a worrisome toxin expression profile and (ii) arise from the hospital environment, especially APHMB rooms, linen and materials, or simply from donating mothers. The toxin gene patterns from various *B. cereus* strains have been studied and seem to be influenced by their geographical and/or foodstuff origins [9]. It was hypothesized that toxin gene patterns could also help us in discerning *B. cereus* strains originating from human milk and the environment. Hence, the aims of this work were first to evaluate the pathogenic potential of strains isolated from human milk and then to compare toxin expression profiles in order to determine if the *B. cereus* contamination of pasteurized human milk in APHMB could arise from environmental strains or not. In a further attempt to differentiate between strains isolated from breast milk and those from the hospital environment, the results obtained were compared to those gathered through analysis of Fourier-transform infra-red (FTIR) spectra, a promising new method for the discrimination of bacterial isolates of the same species [15].

## 2. Materials and methods

### 2.1. Bacillus cereus strain collection

A total of 63 *B. cereus* strains coming from human milk (HM) donations made to the Centre Hospitalier Universitaire APHMB were collected during a previously described study [3]. The study design was validated by the *ad hoc* Research Commission at the Centre Hospitalier Universitaire Amiens-Picardie (institutional review board) following the French Regulation [16]. In accordance with the European General Data Protection Regulation [17], the opinion of the Research Commission on the registration of the databank built for this study by the national commission in charge of data protection (Commission Nationale de l'Informatique et des Libertés) was also sought. The registration was deemed unnecessary (decision date: 24 April 2019).

Twenty-seven environmental strains were retrieved from samples routinely collected through the hospital environment surveillance program. Those included samples coming from APHMB rooms (13 isolates) as well as other wards (4 isolates), linen (7 isolates) and endoscopes (3 isolates).

All strains were identified using matrix-assisted laser desorption ionisation-time of flight mass spectrometry (MALDI Biotyper 2.2; Bruker Daltonik GmbH, Bremen, Germany). They were kept at –20 °C on cryobeads (Mast Diagnostic, Amiens, France) until use.

Additionally, for the detection of toxin genes, several collection strains were included in the analysis as controls: *B. cereus* DSM 31, *B. cereus* DSM 4312 and *B. cereus* DSM 4313, (Deutsche Sammlung für Mikroorganismen und Zellkulturen, Braunschweig, Germany).

### 2.2. PCR detection of toxin genes

DNA extraction was performed using DNeasy Tissue extraction kit (Qiagen, Hilden, Germany) according to the manufacturer's instructions for Gram positive bacteria. Following the extraction procedure, DNA contents in extracts were measured using the Nanodrop apparatus (Thermo Fisher Scientific, Illkirch, France). Amplification of *16S rDNA* (positive control), *ces*, *bceT*, *cytK*, *nhe(ABC)*, and *hbl(ACD)* genes was performed with a Verity thermal cycler (Applied Biosystems, France) using primers previously described (Table 1). A 25 µL reaction volume consisting of 12.5 µL DreamTaq PCR Master Mix (Thermo Fisher Scientific), 1µL of forward and 1 µL of reverse primer (final concentration 0.2 to 0.5 µM), 4.5 µL molecular biology grade water and 5 µL of template DNA was submitted to amplification. The amplification typically consisted of 1 cycle at 94 °C for 5 min followed by 35 cycles including 1 min at 94 °C, 1 min at the mentioned annealing temperature and 2 min at 72 °C. A final elongation cycle at 72 °C for 5 min completed the amplification run. For *ces* amplification, the PCR protocol was composed of a denaturation step at 95 °C for 15 min followed by five cycles of 1 min at 95 °C, 75 s at 53 °C, and 50 s at 72 °C and then by 25 cycles of 1 min at 95 °C, 75 s at 58 °C, and 50s at 72 °C. A final elongation step consisting of 72 °C for 5 min ended the amplification procedure.

**Table 1.** Primer sequences and annealing conditions used in this study.

Target gene	Sequence (5′-3′)	Annealing temperature (°C)	Amplicon size (bp)	Reference
*16S rDNA*	F^a^: ACTCCTACGGGAGGCAG R^a^: ATTACCGCGGCTGCTGGCA	55	196	[18]
*bceT*	F: CGTATCGGTCGTTCACTCGG R: GTTGATTTTCCGTAGCCTGGG	55	661	[19]
*ces*	F: GGTGACACATTATCATATAAGGTG R: GTAAGCGAACCTGTCTGTAACAACA	53/58	1271	[20]
*cytK*	F: CGACGTCACAAGTTGTAACA R: CGTGTGTAAATACCCCAGTT	54	565	[21]
*entFM*	F: GTTCGTTCAGGTGCTGGTAC R: AGCTGGGCCTGTACGTACTT	54	486	[21]
*hblA*	F: AAGCAATGGAATACAATGGG R: AGAATCTAAATCATGCCACTGC	56	1154	[19]
*hblC*	F: GATAC(T,C)AATGTGGCAACTGC R: TTGAGACTGCTCG(T,C)TAGTTG	58	740	[19]
*hblD*	F: ACCGGTAACACTATTCATGC R: GAGTCCATATGCTTAGATGC	58	829	[19]
*nheA*	F: TAAGGAGGGGCAAACAGAAG R: TGAATGCGAAGAGCTGCTTC	54	759	[21]
*nheB*	F: CAAGCTCCAGTTCATGCGG R: GATCCCATTGTGTACCATTG	54	935	[21]
*nheC*	F: ACATCCTTTTGCAGCAGAAC R: CCACCAGCAATGACCATATC	54	618	[21]

^a^: F=Forward primer/R=Reverse primer Amplification products were visualized by electrophoresis on a 1.5% agarose gel containing GelRed^©^ nucleic acid stain (Merck Millipore, Molsheim, France) and run for 1h at 100V followed by UV transillumination on the IBright 1500^©^ system (Thermo Fisher Scientific). Their size was estimated using Generuler DNA ladder (Thermo Fisher Scientific).

### 2.3. Strain typing using Fourier-transform infra-red (FTIR) spectroscopy

All *B. cereus* strains were handled similarly to minimize differences in the carbohydrate, lipid and protein structures of the cell wall that might arise from variations in culture conditions. Frozen strains were subcultured twice on plate count agar (Biomérieux, Marcy l'étoile, France) for 18 to 24 hours at 36 ± 1 °C. Well individualized colonies were then processed using IR Biotyper R^©^ kit (Bruker Daltonik GmbH) according to the manufacturer's instruction. Three replicates of each strain were submitted to FTIR analysis using the IR-Biotyper^©^ system (Bruker Daltonik GmbH) in transmission mode in the spectral range of 4,000–500 cm^–1^ (mid-IR). For each run, quality control was performed with the InfraRed Test Standards (IRTS 1 and 2) in the IR Biotyper R^©^ kit. Resulting spectra were analyzed using OPUS software V8.2.28 (Bruker Daltonik GmbH). Principal Component Analysis (PCA) was applied for multivariate analyses, using the IR Biotyper R^©^ V3.1 software functionality.

### 2.4. Statistical analysis

Comparison of the prevalences of toxin genes and toxin profiles between environmental and HM *B. cereus* strains was performed using Fisher exact test. A p-value below 0.05 was considered as significant.

## 3. Results

### 3.1. Prevalence of toxin gene detection in human milk and environmental *Bacillus cereus* strains

**Figure 1.**
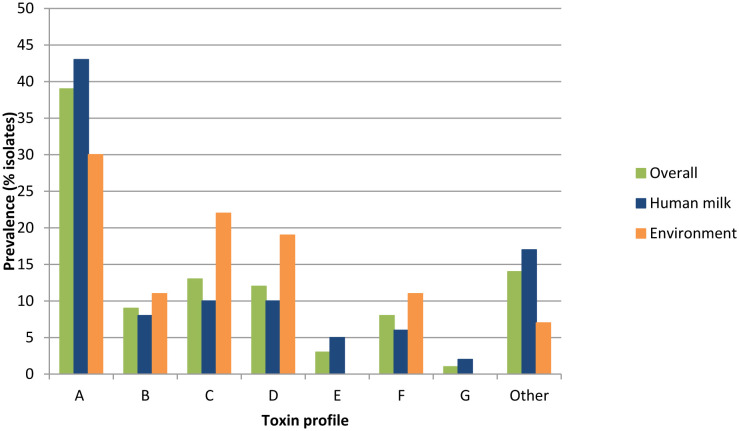
Toxin profiles of *Bacillus cereus* strains included in this study. Toxin profiles are defined as follows: A = *nhe*+, *hbl*+, *cytK*+; B = *nhe*+, *cytK*+, *ces*+; C = *nhe*+, *hbl*+; D = *nhe*+, *cytK*+; E = *nhe*+, *ces*+; F = *nhe*+; G = *cytK*+ and Other = combination of genes different from those described for A to G profiles.

PCR efficiency was first checked for all DNA extracts with the amplification of a control gene (*16S rDNA*). All extracts gave positive results, validating the absence of inhibitory substances that might impair the amplification process. The prevalence of toxin genes and toxin profiles [16] are summarized in Table 2 and Figure 1, respectively. Even though no statistically significant differences in toxin gene prevalences were found between HM and environmental *B. cereus* isolates, prevalences of *nheA, nheB*, and *ces* in HM isolates failed to be qualified as higher than that of environment strains by a narrow margin (p = 0.054; p = 0.063 and p = 0.12, respectively; Fisher exact test). Similarly, no differences in toxin profiles could be highlighted by the statistical analysis comparing HM and environmental isolates.

**Table 2.** Prevalences of toxin genes in *Bacillus* cereus strains included in this study.

Toxin genes	Prevalences		
	Overall (90)^b^	Human Milk (63)	Environment (27)
*bceT*	38 (34)	38 (24)	37 (10)
*ces*	27 (24)	32 (20)	15 (4)
*cytk*	73 (66)	76 (48)	67 (18)
*entFM*	92 (83)	92 (58)	93 (25)
*hblA*	30 (27)	33 (21)	22 (6)
*hblC*	29 (26)	32 (20)	22 (6)
*hblD*	59 (53)	62 (39)	52 (14)
*nheA*	86 (77)	90 (57)	74 (20)
*nheB*	93 (84)	97 (61)	85 (23)
*nheC*	90 (81)	92 (58)	85 (23)

^a^: results expressed as percentage (number of positive isolates) ^b^: number of isolates per category

### 3.2. FTIR analysis of human milk and environmental B. cereus isolates

**Figure 2.**
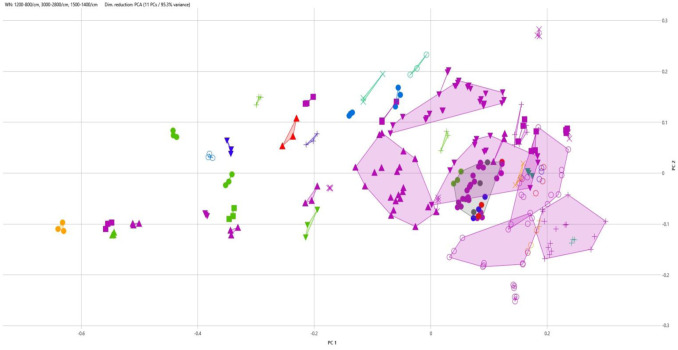
2D plot of the first two components arising from the primary component analysis comparing the 90 *B. cereus* strains included in this study (3 FITR spectra acquired per strain). Color coding for *B. cereus* isolates as follows: 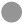 Raw Human Milk; 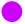 Pasteurized Human Milk; 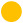 Endoscope; 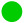 Linen; 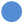 Human Milk Bank Room 1; 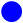 Human Milk Bank Room 2; 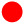 Human Milk Bank Room 3; 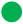 Other hospital wards. Shape coding of the 7 *B. cereus* main (5 isolates and more) clusters as follows: Lower ○ Cluster 1 (5 isolates); Upper ○ Cluster 2 (8 isolates); + Cluster 3 (7 isolates); Upper▼Cluster 4 (7 isolates); Lower ▼Cluster 5 (7 isolates); ▲Cluster 6 (8 isolates); ● Cluster 7 (11 isolates).

A scatter-plot of PCA results from the 90 analyzed strains is displayed in Figure 2. A resulting calculated cut-off distance between isolates of 0.161 was set by the software to discriminate clusters of *B. cereus* isolates. A total of 33 clusters were thus obtained with 18 clusters containing a single isolate, 8 clusters containing either 2 or 3 isolates (4 clusters each). The largest clusters held 5 (1 cluster), 7 (3 clusters), 8 (2 clusters) and 11 (1 cluster) isolates, respectively.

## 4. Discussion

*B. cereus* is a facultative anaerobic, Gram-positive, spore-forming opportunistic pathogen. It is a common cause of food poisoning, the most usual symptoms of which are diarrheal and emetic [8]. One of its prominent virulence features is its ability to produce various toxins. Surprisingly, while plenty of literature can be found on the prevalence and distribution of toxin genes in *B. cereus* strains isolated from cow milk, dairy products and powdered milks, very few works report on these parameters in HM isolates. In the only study found available on the subject, forty-nine isolates (including 3 strains recovered post-pasteurization) were tested for the occurrence of toxin genes [22].

Similar to the high prevalence witnessed in our work, all three *nhe* genes were nearly systematically detected in all but one isolates by these authors. High prevalences ranging from 78 to 100% were also reported for *nhe* in *B. cereus* strains isolated from other raw/pasteurized milk sources and fit with the values found in this work [23]–[27]. As for our *hbl* and *cytK* prevalences, they also fell within the ranges previously described in milk isolated (2 to 90% and 46 to 73%, respectively) [23],[25]–[27]. Regarding *entFM* and *bceT* presence in milk isolates, prevalences were less widely investigated in the literature. While our high *entFM* prevalence was in accordance with the ones already described [24],[25], the 24% prevalence witnessed for *bceT* was lower than those previously published [25],[27]. Finally, *ces* prevalence was null or very low in most previous works reporting on milk isolates [22]–[24],[26] when *ces* prevalence was higher (27%) in the isolates investigated here. Only two studies reported high prevalences for *ces* (10 and 16%, respectively) [25],[27] but none as high as the one found in this work, which is worrisome and supports the current French policy of discarding pasteurized milk displaying a bacterial contamination of 2 Colony Forming Units per milliliter or above [28]. This discrepancy in observed *ces* prevalences might be due to the fact that, as very few *B. cereus* were identified pre-pasteurization in this study, most of the isolates came from pasteurized milk. Indeed, Radmehr *et al*. already noted that isolates harvested post-pasteurization displayed more virulence genes than raw milk ones [26]. It could also be due to the spread of a *B. cereus* clone carrying *ces* in APHMB donations and environment.

As for using toxin gene profiles to discriminate between our isolates, no statistically significant difference in toxin prevalences could be highlighted between environmental and HM isolates investigated in this work. However, *ces* could once more be qualified as concerning as its prevalence was close to being statistically higher in HM isolates than the prevalence in environmental ones. Nevertheless, according to the classification previously described by Ehling-Schulz *et al*., no discriminating pattern between environmental and HM isolates could be identified [14]. However, it must be underscored that this classification did not take into account strains carrying *nhe*, *hbl*, *cytK* and *ces*, nor did it include *entFM*. As a consequence, 13 of our isolates could not be assigned to a given toxin gene profile. We therefore sought another possibility to discriminate between our isolates, such as FTIR spectroscopy.

FTIR spectroscopy has previously been described as being able to discriminate clones between strains of a given species in a time and cost-efficient manner both for Gram-negative and Gram-negative bacteria [15],[29]–[30]. It has also been reported as a helpful tool in discriminating *B. cereus* isolates [26]. The golden standard for discriminating bacterial isolates within a species is the whole genome sequencing (WGS) technique [15]. This method has allowed to drastically improve the discriminatory power over other molecular-based typing methods such as 16S rDNA sequencing, Pulse Field Gel Electrophoresis (PFGE) or Multilocus Sequence Typing (MLST) [15]. However, these molecular-based typing methods are universally recognized as time-consuming, costly and labor-intensive [31]. Several studies have described the interest of spectroscopic/spectrometric-based methods such as Matrix-assisted laser desorption ionization–time of flight mass spectrometry (MALDI-TOF MS) and FTIR in typing bacterial isolates. A major advantage of these techniques is that, once the apparatus is present in a facility, they are easily integrated in the laboratory daily workflow. While MALDI-TOF MS is now routinely used for species identification in medical laboratories, its discrimination power when it comes to clonality assessment has been questioned [31],[32]. The choice of using FITR was therefore made for this work.

Applied to our panel of isolates, FTIR showed that around 42% (38 isolates out of 90) were classified in clusters consisting of a single or 2 to 3 isolates. Hence, no common origin on which a corrective action might be implemented to mitigate the spread of *B. cereus* in pasteurized HM could be identified for those isolates. Nevertheless, more than half of our isolates were grouped in seven clusters of 5 or more isolates (Figure 2). Two of those (Clusters 4 & 6) were only constituted by HM isolates while in the five remaining ones, at least one isolate arose from the hospital environment. Interestingly, in clusters 2 (8 isolates) and 7 (11 isolates), isolates from environmental samples belonging to APHMB rooms were found to aggregate with HM ones. When a closer look was taken at the time sequence in which clusters 2 & 7 isolates were recovered, we found in cluster 7 that the only *B. cereus* strain isolated in raw HM was the first one to be recovered along with its post-pasteurization counterpart and in cluster 2, it was a pasteurized HM isolate. According to FTIR spectra analysis, similar isolates were thereafter harvested from all 3 APHBM rooms in cluster 7 and 2 out of the three APHBM rooms in cluster 2, pointing out that an environmental contamination by the HM donation of one mother could have spread to the environment and other HM donations. It is noteworthy that the first post-pasteurization isolate in cluster 7 was positive for *ces* but only one environmental isolate in the same cluster carried *ces*. Also of interest is the fact that this link between an environmental isolate and some HM ones found in cluster 7 was also highlighted in a previous analysis using rep-PCR [3]. However, this latter method is less suited to a routine determination of isolates' proximity as it is much more time-consuming and costly as well as less discriminating than FTIR spectroscopy. In our case, a closer monitoring of the environment and systematic real-time use of FTIR spectroscopy on *B. cereus* isolates could have helped in limiting the spread of this clone in APHBM as well as the one identified in cluster 2 for which a similar pattern. It might therefore be interesting to implement such a monitoring routinely. Comparing the results obtained through this technique with those of WGS would also help strengthening the daily use of FITR if a good correlation for clonality assessment is found.

## 5. Conclusions

This study is one of the first papers reporting on toxin gene prevalences in HM *B. cereus* isolates and highlights that about one fourth of those are expressing cereulide toxin, which could be worrisome from a public health point of view and call for preventative measures. It is also one of the first reports on *B. cereus* clustering through FTIR Spectroscopy, which proved efficient, time and cost-effective. The use of this technique pointed out that some HM *B. cereus* isolates clustered with APHMB environmental isolates. This is an indication that mitigation measures such as a thorough cleaning procedure following several occurrences of *B. cereus* isolation in a short time span and/or *B. cereus* detection in routine environmental samples could be implemented to reduce the isolation of *B. cereus* in HM donations and the rejection of thusly contaminated donations.
